# Carotenoids synthesis affects the salt tolerance mechanism of *Rhodopseudomonas palustris*

**DOI:** 10.3389/fmicb.2023.1292937

**Published:** 2023-11-22

**Authors:** Meijie Li, Tongtong Zhu, Rumeng Yang, Zhaobao Wang, Min Liu, Jianming Yang

**Affiliations:** ^1^Energy-rich Compound Production by Photosynthetic Carbon Fixation Research Center, Shandong Key Lab of Applied Mycology, College of Life Sciences, Qingdao Agricultural University, Qingdao, China; ^2^State Key Laboratory of Microbial Technology, Shandong University, Qingdao, China

**Keywords:** salt-tolerant, *Rhodopseudomonas palustris*, carotenoid, lycopene, rhodopin

## Abstract

*Rhodopseudomonas palustris* CGA009 is a Gram-negative, purple non-sulfur, metabolically diverse bacterium with wide-ranging habitats. The extraordinary ability of *R. palustris* to decompose a variety of raw materials and convert them into high-value products makes it an attractive host for biotechnology and industrial applications. However, being a freshwater bacterium *R. palustris* has limited application in highly-saline environments. Therefore, it is of great significance to obtain the salt-tolerant strain of *R. palustris* and understand its tolerance mechanism. In this study, *R. palustris* CGA009 was successfully evolved into eight salt-tolerant strains using an adaptive laboratory evolution technique. RPAS-11 (*R. palustris* anti-salt strain 11) was selected as the best salt-tolerant strain and was used in further studies to explore the salt-tolerance mechanism. The expression of most genes associated with the carotenoid synthesis in RPAS-11 increased significantly under high concentration of salt stress, suggesting that carotenoid synthesis is one of the reasons for the salt tolerance of RPAS-11. Gene overexpression and knockout experiments were performed to get clear about the role of carotenoids in salt stress tolerance. RPAS-11-IDI, the mutant with overexpression of IDI (Isopentenyl diphosphate isomerase) exhibited enhanced salt tolerance, whereas the knockout mutant CGA009-∆*crtI* showed a decline in salt tolerance. In addition, the results indicated that rhodopin, a carotenoid compound, was the key pigment responsible for the salt tolerance in *R. palustris*. Furthermore, the production of lycopene, a widely-used carotenoid, was also increased. Taken together, our research helps to deepen the understanding of the salt tolerance mechanism of *R. palustris* and also widens the application of *R. palustris* in highly-saline environments.

## 1 Introduction

*Rhodopseudomonas palustris* is a Gram-negative, purple non-sulfur bacterium (PNSB). Its ability to utilize versatile carbon sources and to flexibly switch between photoautotrophic, photoheterotrophic, chemoheterotrophic, and chemoautotrophic modes of metabolism, makes it a metabolically diverse and environmentally widespread bacterium. It has been isolated from different anaerobic water environments such as lakes, soils, swamps, and oceans (Larimer et al., 2004; Guan et al., 2017). *Rhodopseudomonas palustris* has excellent degradation and detoxification ability, which enables it to degrade lignin, aromatic compounds, insecticides, herbicides and assimilate heavy metals (Li et al., 2022a). Numerous studies are available on the application of *R. palustris* in wastewater treatment, environmental remediation, biofuel production, agricultural biological stimulation, and bioelectricity production (Harwood et al., 1998; Sakpirom et al., 2017; Wu et al., 2019; Li et al., 2022a). Most of these wastes, such as effluents from organic peroxides production, pharmacy, tanneries (1–10%), textiles (3–10%), seafood processing (1.3–3.9%), and petroleum refineries (sometimes higher than 10.5%), exhibit high salt (mainly NaCl) concentrations (Woolard and Irvine, 1995; Díaz et al., 2002; Lefebvre et al., 2005; Lefebvre and Moletta, 2006; Lyles et al., 2008; Xiao and Roberts, 2010; Ogugbue et al., 2011; Amin et al., 2014). However, the low salt tolerance of freshwater *R. palustris* makes it difficult to grow in salt-containing media, which will limit its practical application in high-salinity wastewater treatment. The ability to utilize diverse carbon sources and produce multiple organic compounds makes *R. palustris* an excellent chassis organism for biotechnological research. The bacterium produces not only different biofuels like hydrogen (Zhu X. et al., 2011), methane (Fixen et al., 2016), and butanol (Doud et al., 2017), but also produces high-value compounds, such as terpenoids (Xu et al., 2016), poly-β-hydroxybutyrate (PHB; Wu et al., 2012), and 5-aminolevulinic acid (ALA; Nunkaew et al., 2018). Almost all fermentation processes require sterilization to reduce microbial contamination, which consumes a lot of energy and money. Seawater (contains about 3.5% NaCl; Pernetti and Di Palma, 2005) has a high osmotic pressure, and proper adjustment of its pH can greatly reduce the microbial contamination, which makes it possible to construct a non-sterile open system (Li et al., 2014). A variety of salts in seawater have been shown to facilitate the decomposition of some refractory organics, and a large number of nutrients in polluted seawater can also provide carbon sources for bacteria (Fang et al., 2015; Adessi et al., 2016). At present, there have been many examples of successful fermentation using seawater (Li et al., 2014; Yue et al., 2014; Yu et al., 2019; Meng et al., 2022). Therefore, the utilization of seawater as the substrate for fermentation can be a promising substitute (Pernetti and Di Palma, 2005).

There are two researching directions to realize fermentation in culture media with high concentrations of salt to avoid strict sterile conditions. One researching direction is to engineer the halophilic archaea which can grow optimally with high salt concentrations to produce valuable compounds such as carotenoids, PHBs, and proteins(Giani et al., 2021; Simó-Cabrera et al., 2021). The other researching direction is to evolve the freshwater strains artificially, like *Escherichia coli*, which has been widely utilized as a microbial cell factory (Wu et al., 2014; Adams et al., 2023). *Rhodopseudomonas palustris*, with the properties of extraordinary metabolic versatility, carbon source diversity and metabolite diversity, has been widely applied in wastewater treatment and bioremediation, and it also showed high potential in valuable chemical production, such as lycopene (Li et al., 2022a,b). Our group has focused on valuable chemical production by engineered *R. palustris* through photosynthetic pathways using CO_2_ as the carbon source for 6 years. To widen the application of *R. palustris*, to obtain the salt-tolerant strain and study the mechanism are required.

Most of the reported *R. palustris* strains are not salt-tolerant, with *R. palustris* 42OL as one of the exceptions. Adessi et al. reported that the accumulation of trehalose as a compatible solute protected the bacterial enzyme functions against salt stress, enabling the growth of *R. palustris* 42OL in the salt-amended medium (4.5% salt) without the addition of any exogenous osmoprotectants. Additionally, the production of hydrogen was noticed even with a 3% salt concentration (Adessi et al., 2016). Moreover, the nutrient starvation greatly improved the tolerance of *R. palustris* CGA009 to high-concentration salt treatment (2.5 M NaCl for 1 h), indicating that ATP-dependent rearrangement of cell components can induce salt tolerance (Wasai et al., 2018). These findings build up the understanding of the salt tolerance mechanism of *R. palustris*. However, salt-tolerant *R. palustris* strain has rarely been obtained, and little is known about its salt tolerance mechanisms.

In the present study, salinity evolved *R. palustris* strains were obtained using the adaptive laboratory evolution technique. Based on the salt tolerance analysis RPAS-11 was selected to further explore the salt-tolerant mechanism. In comparison with the wild-type *R. palustris* CGA009, significant color variation was evident in RPAS-11. To understand the color change, the relationship between carotenoid production and salt tolerance in *R. palustris* was evaluated. In addition, the effect of salt stress on lycopene production was also investigated.

## 2 Materials and methods

### 2.1 Microbial strains and cultivation conditions

*Rhodopseudomonas palustris* CGA009 (Kim and Harwood, 1991) was chosen as the original strain for adaptive laboratory evolution (ALE) and the construction of knockout/overexpression strains. *E. coli* strain DH5α was used for gene cloning and strain S17-1 for conjugation. All *E. coli* strains were cultivated in LB (Luria-Bertani) medium supplemented with appropriate antibiotics at 37°C, providing 220 rpm constant shaking. The defined mineral medium (photosynthetic medium, PM) was prepared in accordance with the published article (Brown et al., 2020) for the growth of *R. palustris* strains. A series of *R. palustris* strains were grown anaerobically in PM medium at 30°C, using a 60 W incandescent lamp (General Electric) which was kept on for 24 h a day. Appropriate carbon sources and antibiotics were provided. The salinity of the medium was represented by % w/v, simplified as %, which was the measure of NaCl weight (g) per 100 mL solution. The calculation formula of generation time is: G = lg2* (t2-t1)/lg (x2)-lg (x1). G, the generation time (h); t2 and t1, culture time (h); x2 and x1, OD_660_ corresponding to culture time t2 and t1 (Anderson et al., 2021).

### 2.2 Adaptive laboratory evolution

To obtain strains with better salt tolerance, ALE was performed. PM liquid medium supplied with 20 mM NaAc (sodium acetate) was used as the basic medium. At first, *R. palustris* CGA009 was cultured in 10 mL basic medium. Then the bacterial medium in the logarithmic phase was transferred into the basic medium supplied with 1.5% salt (NaCl), and two generations of cultivation at the same salt concentration were performed. Then, the salt concentration of the medium was gradually increased by 0.2% and the salt concentration was increased to the final 4%. To isolate single colonies, the bacterial culture mixture was spread on plates with 2 × PM solid medium supplemented with 4% salt, 20 mM NaHCO_3_ and 4 mM Na_2_S_2_O_3_·5H_2_O, and the plates were cultivated under anaerobic conditions. Then, the cell growth of the single colonies under different salt concentrations was tested to screen the colonies with increased salt tolerance. At first, the single colonies on the plates were transferred into PM medium supplied with 20 mM NaAc under 1.5% salt concentration, separately. Then, the bacterial medium was transferred into PM medium supplied with 20 mM NaAc under high salt concentrations, including 0, 2.0, 2.5 and 3.0%. The cell growth was monitored and a single colony named RPAS-11 which showed higher cell growth was selected for further research.

### 2.3 Construction of plasmids and strains

Plasmids and strains used in this work were listed in Table 1. *crtC* was amplified from the genome of *R. palustris* CGA009 using primers crtC-F/R, and the vector was amplified using primers pBBRzt-F/R from pBBRMCS-5, and Gibson assembly was applied to construct pBBR-crtC. pBBR-IDIsc (Li et al., 2022b) and pBBRMCS-5 (Kovach et al., 1995) was obtained before. Through conjugation, pBBR-IDIsc was transformed into CGA009 and RPAS-11, obtaining CGA009-IDI and RPAS-11-IDI, respectively, (Wang et al., 2020). Using the same method, the empty plasmid pBBRMCS-5 was transformed into CGA009 and RPAS-11, constructing CGA009-pBBRkz and RPAS-11-pBBRkz, respectively. Also, pBBR-crtC was transformed into CGA009, obtaining CGA009-CrtC. In accordance with the method previously described (Quandt and Hynes, 1993; Egland et al., 1995; Jiao and Newman, 2007), the gene knockout in *R. palustris* CGA009 was carried out. All primers used for construction of suicide plasmid and validation of recombinant strain are listed in Supplementary Table S2. Through conjugation, the constructed plasmid was transformed from *E. coli* S17-1 to target *R. palustris* strain (Egland et al., 1995). Constructed suicide plasmid and recombinant strain are listed in Table 1.

**Table 1:** Plasmids and strains constructed in this work.

Names	Descriptions	References
pBBRMCS-5	Broad-host-range vector, Gm^r^	Kovach et al. (1995)
pJQ200SK	Suicide vector, sacB, Gm^r^	Quandt and Hynes (1993)
pBBR-IDI_sc_	pBBR1MCS-5 carrying *IDI* from *S. cerevisiae* (*IDI_sc_*); Gm^r^	Li et al. (2022b)
pBBR-crtC	pBBR1MCS-5 carrying *crtC* from *R. palustris*; Gm^r^	This study
pJQ200SK-Δ*crtI*	pJQ200KS-based plasmid for *crtI* deletion; sacB; Gmr	This study
pJQ200SK-Δ*crtC*	pJQ200KS-based plasmid for *crtI* deletion; sacB; Gmr	This study
pJQ200SK-Δ*crtD*	pJQ200KS-based plasmid for *crtI* deletion; sacB; Gmr	This study
pJQ200SK-Δ*crtF*	pJQ200KS-based plasmid for *crtI* deletion; sacB; Gmr	This study
*R. palustris* RPAS-11	Screened salt-tolerant strain	This study
CGA009-IDI	*R. palustris* CGA009/pBBR-IDI_sc_	This study
RPAS-11-IDI	*R. palustris* RPAS-11/pBBR-IDI_sc_	This study
CGA009-pBBRkz	*R. palustris* CGA009/pBBRMCS-5	This study
RPAS-11-pBBRkz	*R. palustris* RPAS-11/pBBRMCS-5	This study
CGA009-CrtC	*R. palustris* CGA009/pBBR-crtC	This study
CGA009-Δ*crtI*	*R. palustris* CGA009 *with crtI* disruption	This study
CGA009-Δ*crtC*	*R. palustris* CGA009 with *crtC* disruption	Li et al. (2022b)
CGA009-Δ*crtD*	*R. palustris* CGA009 with *crtD* disruption	Li et al. (2022b)
CGA009-Δ*crtF*	*R. palustris* CGA009 with *crtF* disruption	Li et al. (2022b)

### 2.4 Quantitative real-time PCR (qRT-PCR)

*Rhodopseudomonas palustris* CGA009 and RPAS-11 were cultured at 30°C in PM, supplemented with 20 mM NaAc and various concentrations of salt (0, 1.0 and 2.0%). The cells were harvested in the logarithmic phase with a OD_660_ at 0.8. RNA extraction, reverse transcription, and real-time quantitative PCR were performed according to the procedures reported before (Wang et al., 2020). All primers used for real time qPCR analysis are listed in Supplementary Tables S1, S2.

### 2.5 ROS test

*Rhodopseudomonas palustris* CGA009 and RPAS-11 were cultured at 30°C in PM, supplemented with 20 mM NaAc and various concentrations of salt (0, 1.0 and 2.0%). The cells were harvested in the logarithmic phase and washed with PBS (phosphate buffer saline). ROS was detected using fluorometric probe 2′, 7′-dichlorodihydrofluoresceine diacetate (DCFH-DA), which was dissolved with ethanol and stored at −20°C in dark (Sigma-Aldrich, United States; He and Häder, 2002). The collected was resuspend by DCFH-DA with the final concentration of 10 μM, and kept for 30 min in dark. Excess DCFH-DA was removed by three washes with PBS. The fluorescence of samples was analyzed with an excitation wavelength of 485 nm and an emission band between 500 and 600 nm using an Ultra-sensitive multi-function microchannel plate detector (Biotek, United States; Verma et al., 2019).

### 2.6 Pigment extraction

200 μL cells were collected by centrifugation at 10,000 × g for 2 min at 4°C and rinsed twice with deionized H_2_O to wash the cells. 1 mL of acetone: methanol (1:1 v/v) solution was added into the bacterial pellet to extract the pigment. The suspension was shaking at 30°C for 10 min in the dark, centrifuged at 4°C with 10,000 × g for 2 min. The shaking was repeated until the bacterial pellet was colorless, and the acetone: methanol layer containing the pigment was transferred to a new test tube. Absorption spectrum of the extracted pigments was measured in the range of 100 to 900 nm by a UV/VIS spectrophotometer (Cary 60 UV–Vis, Agilent Technologies, United States).

### 2.7 Quantification of carotenoids

All steps were carried out under dim light. Carotenoids were transferred into hexane by three successive extractions of the acetone: methanol layer. Quantification of total carotenoids (C, mg/g DCW, Dry cell weight) was determined by the following formula (Abidin et al., 2014): C = (D·V·f × (10/2500))/DCW (g), where, D, absorbance at 480 nm; V, total volume of sample used (mL); f, dilution factor of sample. 2,500 is the E1cm1%, namely average extinction co-efficient for carotenoids, and 10 is the factor to convert % to mg/mL. DCW was determined according to the reference (Li et al., 2022b).

### 2.8 HPLC analysis of lycopene in *Rhodopseudomonas palustris*

The content of lycopene was analyzed by HPLC (Agilent Technologies Series 1,200 system, Agilent, United States). The detection wavelength of UV detector was set to 480 nm, and the column used was a symmetrical C18 column (250 mm × 4.6 mm, 5 μm, Waters, Ireland). Methanol/acetonitrile/dichloromethane (21:21:8, volume ratio) was selected as the mobile phase. The flow rate was set at 1 mL/min and the chromatographic column was kept at 30°C. Lycopene standard was purchased from Sigma-Aldrich and dissolved with methylene dichloride. Standard curve of lycopene was obtained by stepwise dilution of the standard and injection analysis, which was used for subsequent yield calculation.

## 3 Results

### 3.1 Screening of salt-tolerant strain through adaptive laboratory evolution

**Figure 1:**
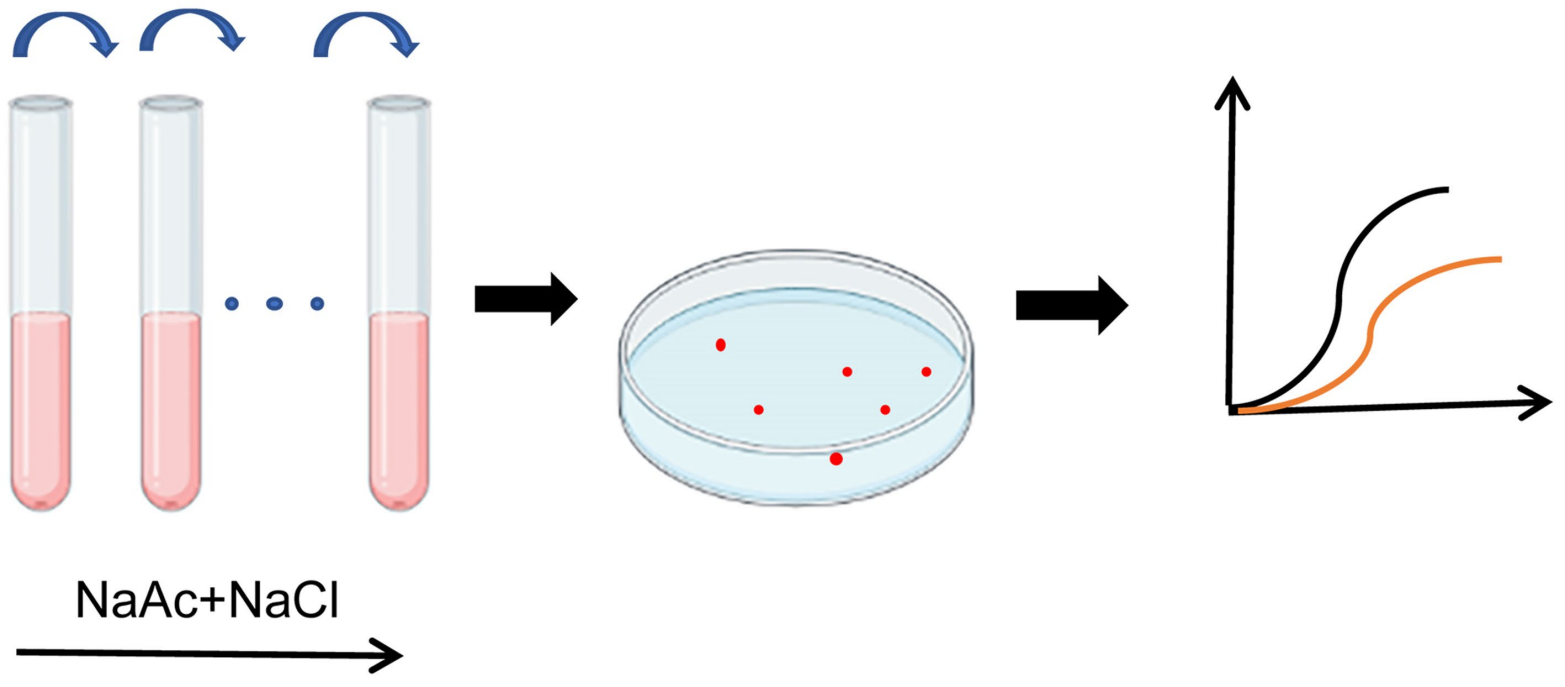
Illustration of the ALE process. *Rhodopseudomonas palustris* CGA009 was anaerobically cultured in 10 mL PM medium at 30°C under the incandescent lamp, using 20 mM NaAc as a carbon source and 1.5% salt concentration. The culture in the logarithmic phase was further transferred to a fresh medium. After every three generations, the salt concentration in the medium was increased by 0.2%. After 8 months of culture, the salt concentration was increased to the final 4% and stabilized for about 10 generations. The single colonies were isolated and screened for salt tolerance by growth curve.

To obtain *R. palustris* strains with salt-tolerant properties, wild-type *R. palustris* CGA009 was transferred into 10 mL liquid PM medium using 20 mM NaAc as a carbon source with increasing salt concentration. Starting with 1.5%, the salt concentration was gradually increased by 0.2% and the salt concentration was increased to the final 4%. After ALE, the bacterial culture was spread plated on a 2 × PM solid medium supplemented with 20 mM NaAc and 4% salt. Single colonies were isolated and inoculated into PM liquid media with 1.5% salt concentration for activation. Bacterial cultures in the logarithmic phase were transferred into PM media supplied with 20 mM NaAc under high concentrations (0, 2.0, 2.5, and 3.0%) of salt. Growth curves were measured to test the salt tolerance of strains and obtain the final salt-tolerant strain (Figure 1).

**Figure 2:**
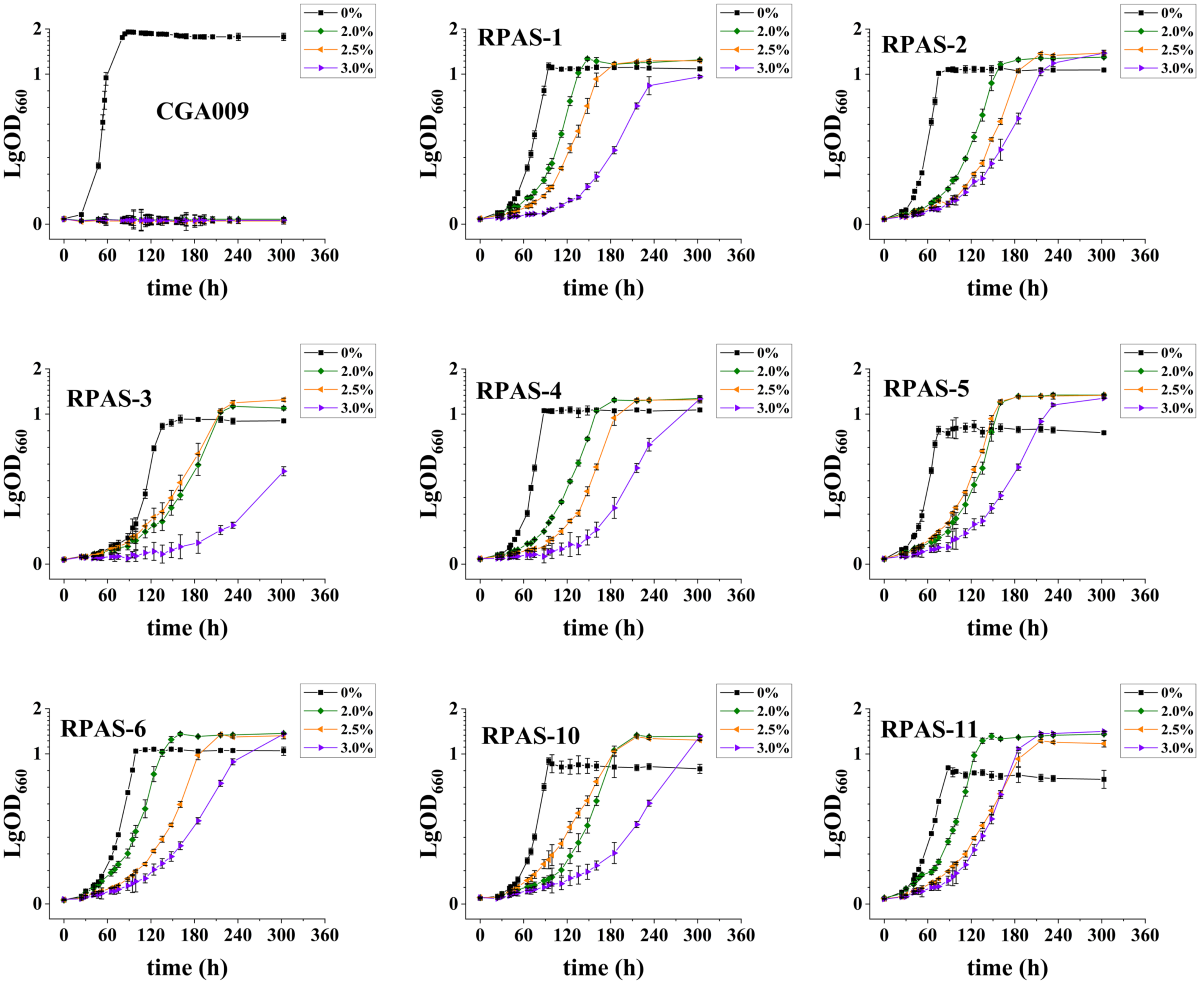
Growth curves of eight screened salt-tolerant *R. palustris* strains. Strains were cultivated in PM medium with 20 mM acetate as the carbon source at different salt concentrations (0, 2.0, 2.5, and 3.0%). Three biological replicates were performed for each treatment.

**Figure 3:**
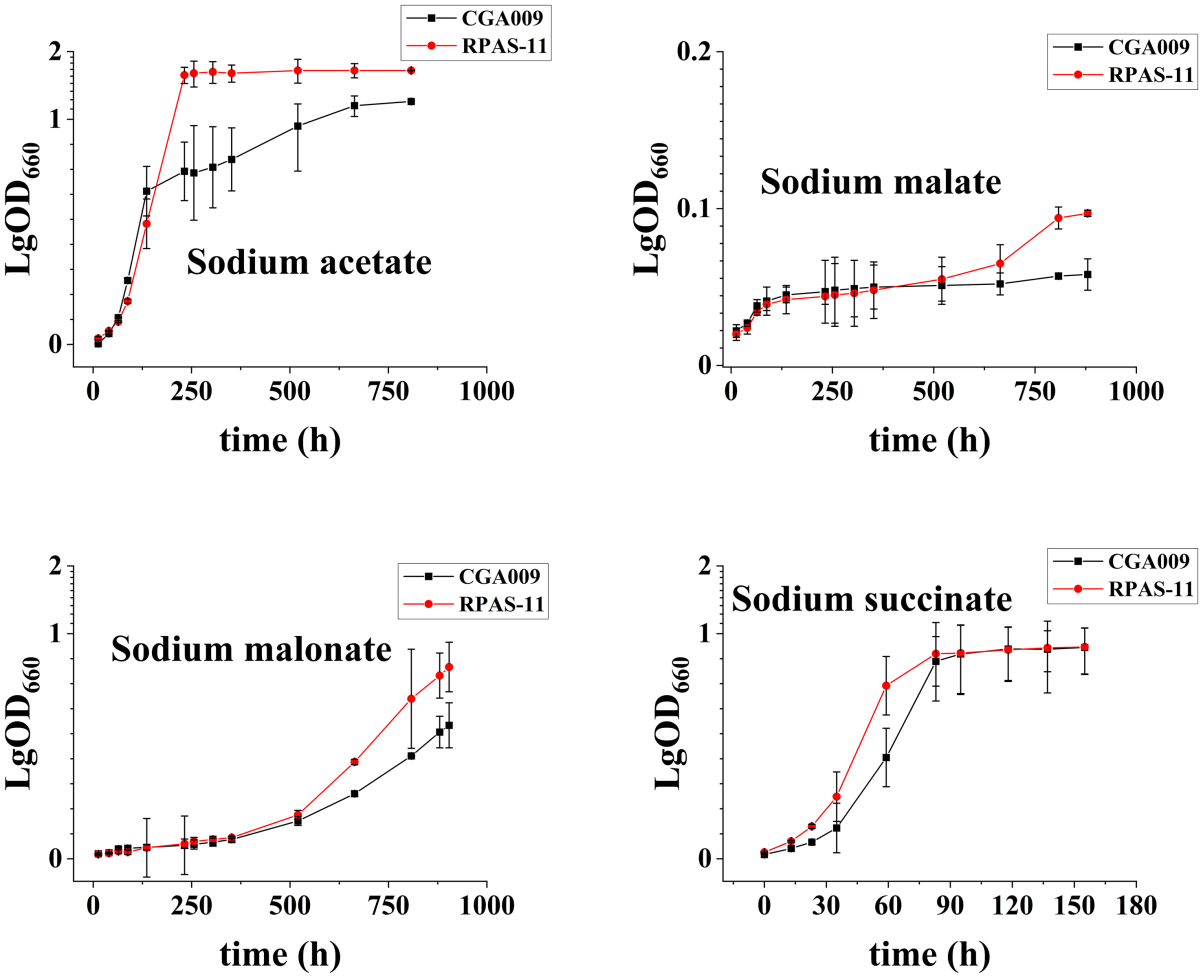
Growth curves of CGA009 and RPAS-11 using different carbon sources. Four carbon sources, including acetate, succinate, malate, and malonate, were selected for CGA009 and RPAS-11 growth with a 1.5% salt concentration. Three biological replicates were performed for each treatment.

Eight salt-tolerant *R. palustris* strains were screened for their growth at different salt concentrations, in comparison to the growth rate of ancestral wild-type CGA009, as represented in Figure 2. Out of eight, RPAS-11 exhibited salt tolerance at all salt concentrations, as indicated by the reduced generation time and optimized growth curve (Figure 2; Table 2). Besides, RPAS-11 also showed growth in a medium containing 4.5% salt concentration, while wild-type CGA009 could hardly grow (Supplementary Figure S1). Seawater contains about 3.5% NaCl, in which many microorganisms are difficult to survive (Pernetti and Di Palma, 2005). The strain we obtained can tolerate up to 4.5% salt concentration, so it is entirely possible to apply it to seawater fermentation. Further, the growth of RPAS-11 and CGA009 was evaluated using four different carbon sources at 1.5% salt concentration (Figure 3). RPAS-11 showed comparatively better growth than that of wild-type CGA009 under salt stress using different carbon sources (Figure 3), which further demonstrated the salt tolerance of RPAS-11 irrespective of carbon source.

**Table 2:** Generation time of CGA009 and eight salt-tolerant *Rhodopseudomonas palustris* strains.

	Generation time(h)			
Salt concentration	0%	2%	2.5%	3.0%
CGA009	12.27 ± 0.11	57.21 ± 0.12	165.28 ± 0.06	183.80 ± 0.19
RPAS-1	17.07 ± 0.21	21.62 ± 0.08	35.61 ± 0.19	37.37 ± 0.13
RPAS-2	15.94 ± 0.09	27.85 ± 0.21	31.43 ± 0.13	44.21 ± 0.14
RPAS-3	28.73 ± 0.10	47.42 ± 0.24	43.92 ± 0.11	53.63 ± 0.19
RPAS-4	14.66 ± 0.12	30.63 ± 0.03	35.68 ± 0.13	37.34 ± 0.21
RPAS-5	16.76 ± 0.12	35.71 ± 0.15	33.23 ± 0.19	52.23 ± 0.02
RPAS-6	17.07 ± 0.22	27.40 ± 0.19	35.82 ± 0.17	43.22 ± 0.05
RPAS-10	18.23 ± 0.16	34.82 ± 0.27	34.81 ± 0.23	46.01 ± 0.14
RPAS-11	15.64 ± 0.18	26.93 ± 0.09	37.84 ± 0.29	32.25 ± 0.19

### 3.2 Carotenoids production and expression of carotenoids synthesis genes under salt stress in CGA009 and RPAS-11

**Figure 4:**
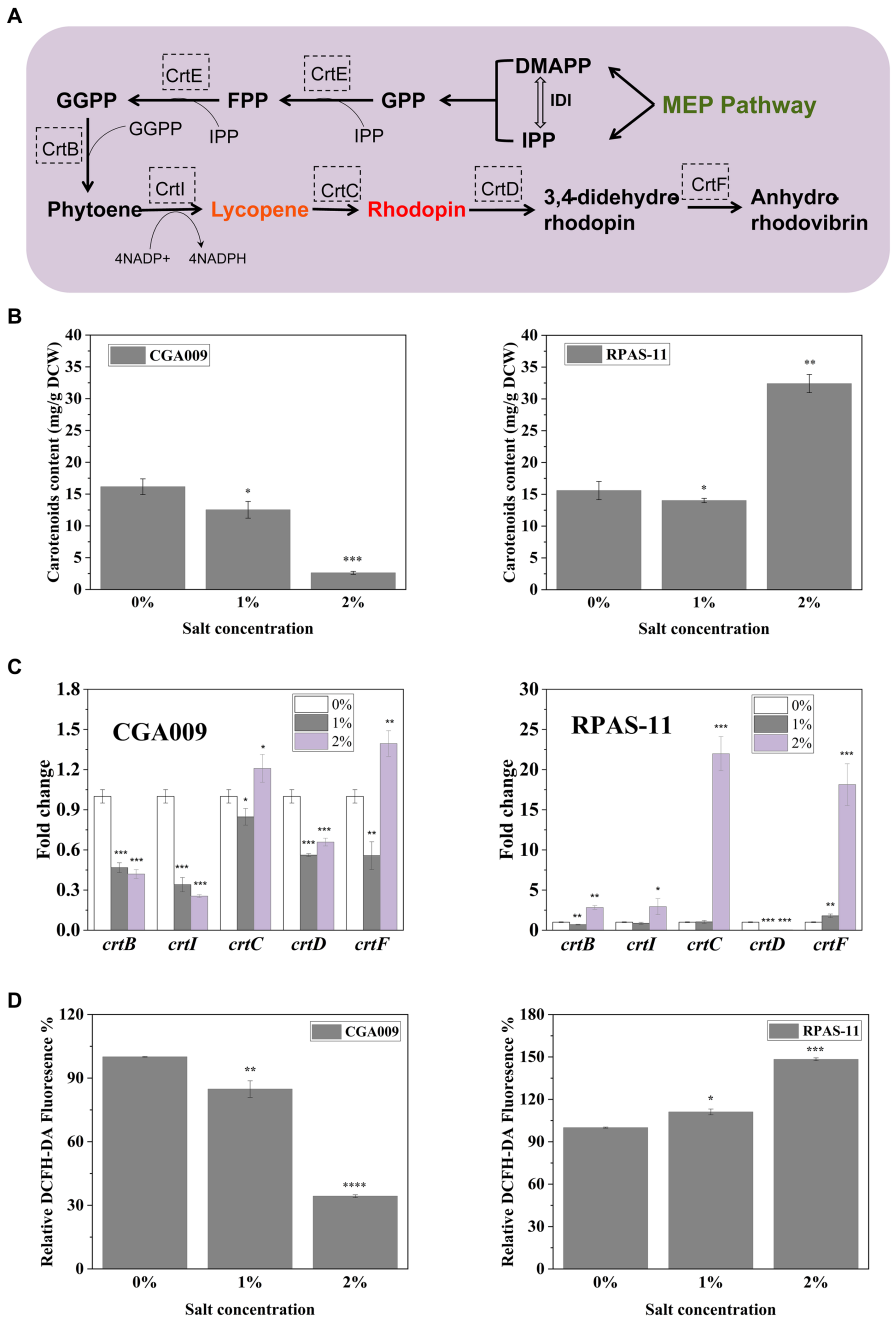
Transcription of carotenoid biosynthesis genes in RPAS-11 and CGA009. **(A)** Illustration of the related carotenoid biosynthetic pathway in *R. palustris*. The endogenous MEP pathway is responsible for the synthesis of precursors DMAPP and IPP. MEP Pathway, Methylerythritol phosphate pathway; IPP, Isopentenyl diphosphate; DMAPP, Dimethylallyl diphosphate; GPP, Geranyl diphosphate; FPP, Farnesyl diphosphate; GGPP, Geranylgeranyl diphosphate; IDI, Isopentenyl-diphosphate isomerase; CrtE, FPP/GGPP synthase; CrtB, Phytoene synthase; CrtI, Phytoene desaturase; CrtC, Hydroxyneurosporene synthase; CrtD, FAD dependent oxidoreductase; CrtF, O-Methyltransferase family **(B)** Carotenoids content of CGA009 and RPAS-11 under different salt concentrations (0, 1, and 2%). **(C)** Real-time quantitative analysis of genes related to carotenoid synthesis in CGA009 and RPAS-11. The transcription level of genes related to carotenoid synthesis at 0% salt concentration was used as the control. **(D)** Relative DCFH-DA fluorescence (%) of ROS produced after NaCl stress. Three biological replicates were performed for each treatment. Error bars: mean ± standard deviation (*n* = 3). *p < 0.05 was considered to be statistically significant. **p < 0.01, ***p < 0.001. *p* value was calculated by Excel.

In comparison to wild-type CGA009, the screened strains displayed variation in colors at different salt concentrations (Supplementary Figure S2). This change in color can be attributed to the change in carotenoid synthesis in the cell membranes of *R. palustris*. CGA009 showed a decrease in carotenoids production with increasing salt concentrations, where 16.15 mg/g DCW and 2.61 mg/g DCW carotenoids were produced at 0 and 2% salt concentrations, respectively (Figure 4B). This result was in concordance with the color change of CGA009 observed under salt stress (Supplementary Figure S2). However, the carotenoids production was increased in RPAS-11 from 15.58 mg/g DCW to 32.40 mg/g DCW with an increase in salt concentration from 0 to 2%, respectively (Figure 4B). It was reasonable to say that the increase in carotenoids production under salt stress might be related to the salt tolerance mechanism of RPAS-11. To further elucidate the mechanism of carotenoids production under salt stress, reactive oxygen species (ROS) levels were measured in CGA009 and RPAS-11 under salt stress (1 and 2%; Figure 4D; Ren et al., 2021). Salt stress increases the production of ROS, which can be eliminated by antioxidants, such as carotenoids. Therefore, under the salt stress, ROS level is directly proportional to carotenoids accumulation. In RPAS-11, increased ROS level and carotenoids production under salt stress were detected (Figure 4D). In CGA009, the ROS value was decreased under salt stress, just as the reduction of carotenoids production under salt stress (Figure 4D). It was speculated that, in wild-type CGA009, salt stress (1 and 2%) might damage the cells metabolism to produce ROS and carotenoids.

No significant difference was evident in the carotenoids content of CGA009 (16.15 mg/g DCW) and RPAS-11 (15.58 mg/g DCW) at 0% salt concentration; however, both the strains displayed different colors (Supplementary Figure S2). In *R. palustris*, carotenoids, including lycopene, anhydro-rhodovibrin, rhodovibrin, rhodopin, and spirilloxanthin are synthesized through the MEP pathway, and the carotenoids synthetic pathway (Figure 4A; Li et al., 2022b). It was indicated that the expression of carotenoid synthetic genes (*crtB*, *crtI*, *crtC*, *crtD*, and *crtF*) might also change under salt stress. To test this speculation, RT-qPCR was performed to determine the effect of different salt concentrations on the transcription of genes associated with the carotenoid synthesis in CGA009 and RPAS-11 (Figure 4C).

In CGA009, the transcriptional expressions of all five genes (*crtB*, *crtI*, *crtC*, *crtD*, and *crtF*) were decreased under 1% salt stress (Figure 4C). Under salt stress, cell metabolism was inhibited, which might weaken the synthesis of carotenoids, showing a decrease in the expression of related genes in the experimental results. However, the expression levels of *crtB*, *crtI* and *crtD* were reduced, whereas *crtC* and *crtF* showed elevated expression at 2% salt stress in comparison to expression without salt stress. In the carotenoids synthetic pathway, enzymes CrtC and CrtF were responsible for rhodopin, anhydrorhodovibrin, rhodovibrin, and spirilloxanthin production (Giraud et al., 2018). Based on this, we speculated that 2% salt stress might stimulate the synthesis of these pigments, helping *R. palustris* to adapt to salt stress in turn.

In the salt-tolerant strain RPAS-11, expression levels of most carotenoid synthesis genes remained unchanged or increased under salt stress (1 and 2%), except *crtD* (Figure 4C). Further, the transcription of *crtB*, *crtI*, and *crtC* was up-regulated by 3-, 3-, and 22-fold, respectively, at 2% salt concentration, in comparison to no salt stress (Figure 4C). Our results indicated that the expression of carotenoid synthesis genes in RPAS-11 was up-regulated under salt stress. Therefore, we speculated that RPAS-11 acquired mutations that increased carotenoid production to combat salt stress. However, the results of RT-qPCR analysis indicated that *crtD* was hardly expressed under salt stress. The enzyme CrtD is responsible for catalyzing the synthesis of 3,4-didehydro-rhodopin from rhodopin (Figure 4A). Therefore, the reduced expression of *crtD* allows RPAS-11 to accumulate rhodopin. These results suggested rhodopin accumulation in salt-tolerant RPAS-11 cells. The expression of *crtF* was also increased under salt stress. Enzyme CrtF is responsible for catalyzing the synthesis of anhydrorhodovibrin from 3,4-didehydro-rhodopin (Figure 4A). If *crtD* was hardly expressed, the expression of *crtF* could not function as usual. The results suggested that the salt-tolerant RPAS-11 could accumulate carotenoids to neutralize the salt stress, and rhodopin might play a crucial role among these carotenoids.

### 3.3 Carotenoid accumulation helps *Rhodopseudomonas palustris* tolerate salt stress

Our results demonstrated that RPAS-11 increases carotenoid production during salt stress, and that rhodopin may be the primary carotenoid that is overproduced. In order to establish the role of carotenoids in the salt tolerance of *R. palustris*, a series of mutant strains with knockout or overexpression of genes related to carotenoid synthesis were constructed, and their salt tolerance was evaluated.

**Figure 5:**
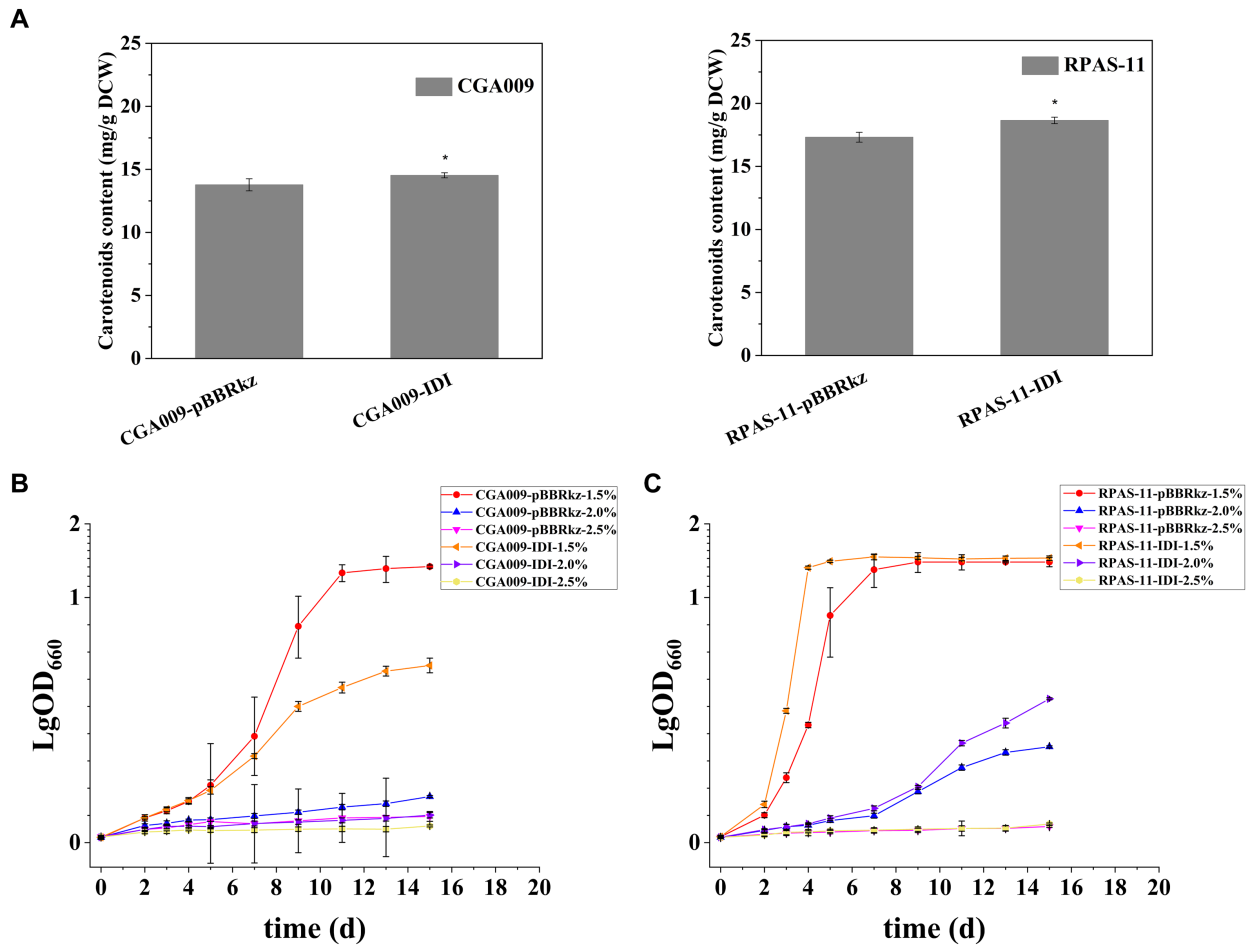
Carotenoids and growth curve after overexpression of IDI in CGA009 and RPAS-11. **(A)** Carotenoids content of CGA009-pBBRkz, CGA009-IDI, RPAS-11-pBBRkz, and RPAS-11-IDI without salt stress. Error bars: mean ± standard deviation (*n* = 3). *p < 0.05 was considered to be statistically significant. p value was calculated by Excel. **(B)** Growth curve of CGA009-pBBRkz and CGA009-IDI at different salt concentrations (1.5, 2.0, and 2.5%). **(C)** Growth curve of RPAS-11-pBBRkz and RPAS-11-IDI with different salt concentrations (1.5, 2.0, and 2.5%). All these strains were cultured in PM medium with 20 mM acetate as the carbon source. Three biological replicates were performed for each treatment.

First of all, the effect of increased carotenoid accumulation on salt tolerance was determined. In *R. palustris*, the precursors of the carotenoids synthetic pathway, isopentenyl diphosphate (IPP) and dimethylallyl diphosphate (DMAPP), are synthesized through the methylerythritol phosphate (MEP) pathway (Figure 4A; Li et al., 2022b). The overexpression of IDI is responsible for catalyzing the reversible conversion of IPP and DMAPP in the MEP pathway (Figure 4A), resulting in increased production of carotenoids, especially lycopene (Li et al., 2022b). Therefore, strains CGA009-IDI and RPAS-11-IDI exhibiting overexpression of IDI were constructed to improve carotenoid production in CGA009 and RPAS-11 (Table 1). Strains CGA009-pBBRkz and RPAS-11-pBBRkz carrying an empty vector pBBRMCS-5 were constructed and used as the controls (Table 1). The IDI overexpression resulted in a slight increase in the total carotenoids content in both CGA009 and RPAS-11 (Figure 5A). Further, the growth of the bacterial strains was analyzed at different salt concentrations (1.5, 2.0, and 2.5%) to determine the salt tolerance. The resultant decline in the growth rate of CGA009-IDI at 1.5 and 2.0% salt concentrations might be due to the consumption of cell energy in IDI overexpression (Figure 5B; Table 3). On the contrary, strain RPAS-11-IDI exhibited a higher value of final OD_660_ irrespective of the IDI overexpression, as compared to RPAS-11-pBBRkz, under different salt concentrations, especially under 2.0% salt concentration, indicating higher tolerance of RPAS-11-IDI (Figure 5C). These results indicated that the increased carotenoids content by overexpression of IDI might be a reason for the improved salt tolerance in strain RPAS-11-IDI.

**Table 3:** Generation time of RPAS-11 and CGA009 strains overexpressing IDI under salt stress.

	Generation time (h)			
Salt concentration	CGA009-pBBRkz	CGA009-IDI	RPAS-11-pBBRkz	RPAS-11-IDI
1.5%	56.58 ± 0.19	68.86 ± 0.12	23.26 ± 0.19	16.29 ± 0.18
2.0%	248.3 ± 0.22	386.7 ± 0.11	105.7 ± 0.23	81.78 ± 0.13
2.5%	447.0 ± 0.23	495.2 ± 0.16	267.8 ± 0.32	249.9 ± 0.25

Symbol ± indicated the standard value.

**Figure 6:**
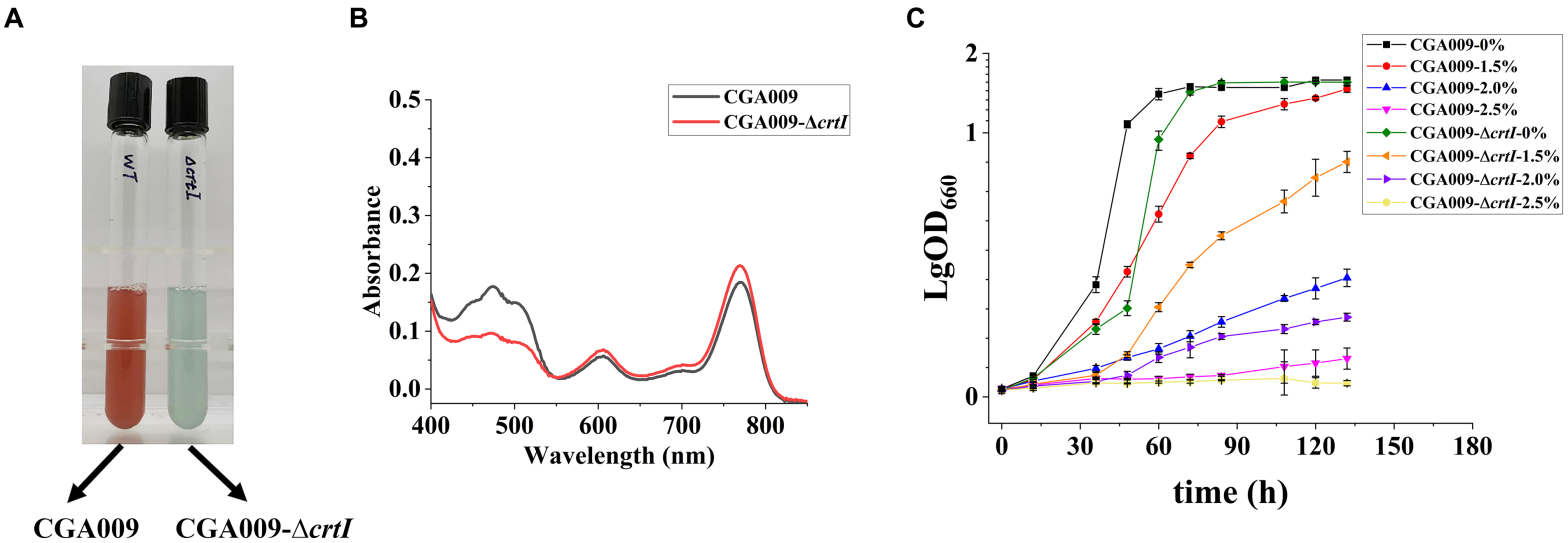
Growth curve and UV–visible absorption spectrum for pigments of CGA009 strains with and without *crtI* deletion. **(A)** The color of strain CGA009 and CGA009-Δ*crtI* cultured in a salt-free environment. **(B)** UV–visible absorption spectra (400–850 nm) of extracted pigments of CGA009 and CGA009-Δ*crtI*. **(C)** Growth curve of CGA009 and CGA009-Δ*crtI* at different salt concentrations (0, 1.5, 2.0, and 2.5%). Strains were cultivated in a PM medium with 20 mM acetate as the carbon source. Three biological replicates were performed for each treatment.

In order to reverse verify the effect of carotenoids on the salt tolerance of *R. palustris*, we studied the salt tolerance of *R. palustris* without carotenoids. In the carotenoid synthetic pathway, enzyme CrtI is known to catalyze the conversion of phytoene to lycopene; therefore, the deletion of gene *crtI* (strain CGA009-Δ*crtI*) would not only inhibit the lycopene production but also block the formation of other carotenoids (Zhu L. et al., 2011; Giraud et al., 2018). The *crtI* knockout strain CGA009-Δ*crtI* displayed green color, which can be attributed to bacteriochlorophyll (BChl; Figure 6A), another light-absorbing pigment present in *R. palustris*. The results of absorption spectra (400–850 nm) of extracted pigments from CGA009 and CGA009-Δ*crtI* indicated that no carotenoids were synthesized in CGA009-Δ*crtI* (Figure 6B). Further, the growth of CGA009-Δ*crtI* and CGA009 was analyzed at different salt concentrations (Figure 6C). Under salt-free condition, the final OD_660_ value of CGA009-Δ*crtI* was similar to CGA009, and the generation time, 11.97 ± 0.18, was slightly higher than that of CGA009, 10.27 ± 0.10 (Figure 6C; Table 4). This result indicated that deletion of *crtI* slightly affected the cell growth. Deletion of *crtI* inhibited the synthesis of carotenoids, which can capture light of the visible spectrum and, then, transfer the excitation energy to BChl (Adessi and De Philippis, 2014). At higher salt concentrations (1.5, 2, and 2.5%), the final OD_660_ values of CGA009-Δ*crtI* were lower than that of wild-type CGA009, and the growth rate was also affected (Figure 6C; Table 4). Under salt stress, the extent of the effect on cell growth was more serious than that under salt-free condition, indicating the weakening salt tolerance of strain CGA009-Δ*crtI*. These results also established the role of carotenoid production in salt stress tolerance. Therefore, it was reasonable to say that the deletion of *crtI* not only disrupted the carotenoid production but also weakened the salt tolerance, thereby confirming the positive effect of carotenoids on the salt tolerance of *R. palustris*.

**Table 4:** Generation time of *crtI* knockout strain and wild-type CGA009 under salt stress.

	Generation time (h)	
Salt concentration	CGA009	CGA009-Δ*crtI*
0%	10.27 ± 0.10	11.97 ± 0.18
2.0%	45.14 ± 0.13	51.80 ± 0.09
2.5%	131.1 ± 0.11	157.5 ± 0.12

Symbol ± indicated the standard value.

**Figure 7:**
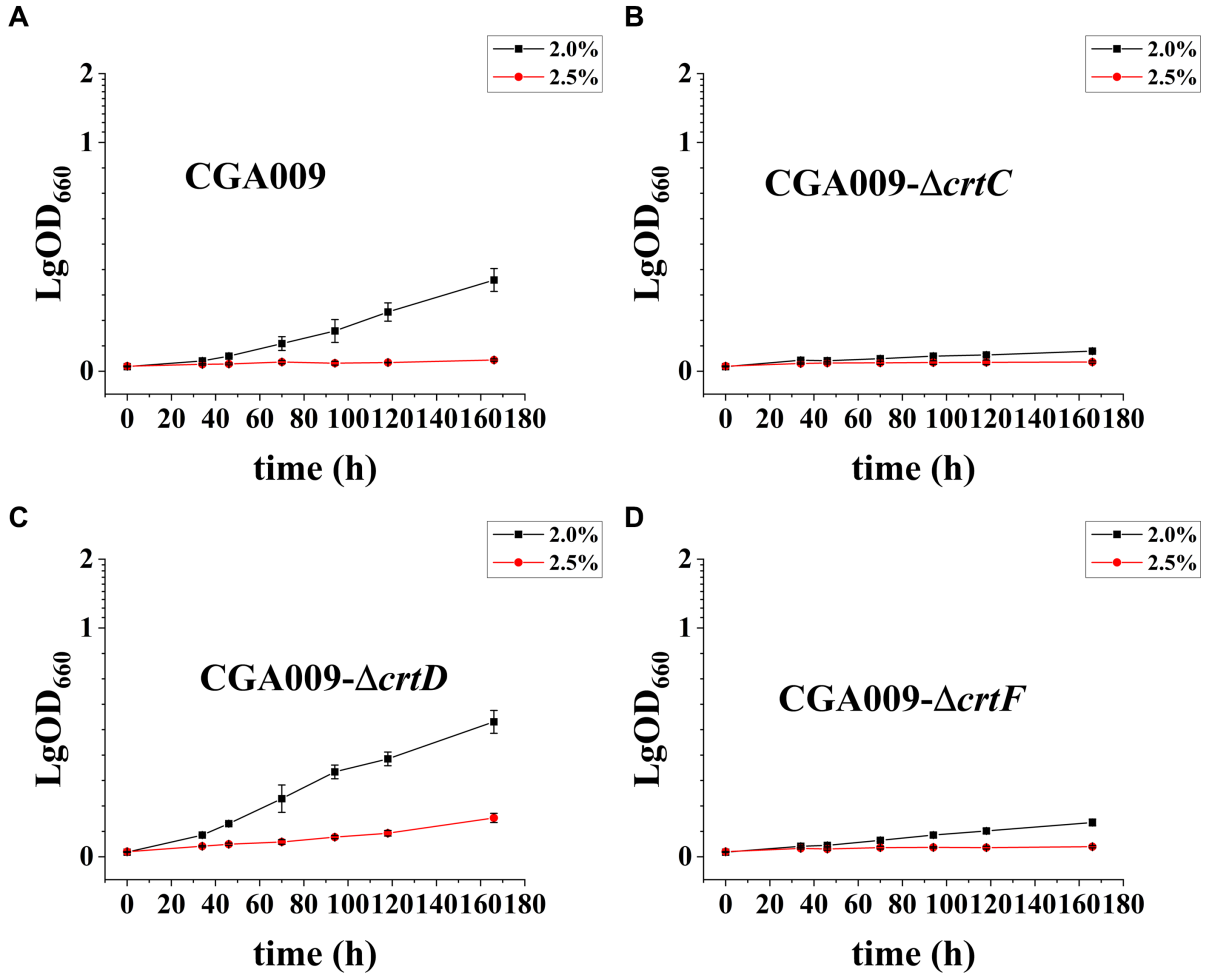
Growth curve of *R. palustris* strains with *crtC*, *crtD*, or *crtF* deletion under salt stress. **(A)** Growth curve of CGA009 under salt stress. **(B)** Growth curve of CGA009-Δ*crtC* under salt stress. **(C)** Growth curve of CGA009-Δ*crtD* under salt stress. **(D)** Growth curve of CGA009-Δ*crtF* under salt stress. Strains were cultivated in a PM medium containing different salt concentrations (2.0 and 2.5%) with 20 mM acetate as the carbon source. Three biological replicates were performed for each treatment.

After demonstrating the effect of carotenoids on salt tolerance, the key pigment among the carotenoids was identified. To determine the key pigment, three carotenoid biosynthetic genes knockout strains, namely CGA009-Δ*crtC*, CGA009-Δ*crtD*, and CGA009-Δ*crtF*, were constructed and evaluated for their growth at different salt concentrations to assess their salt tolerance (Figure 7; Table 5). The results indicated that gene knockout significantly affected the growth of *R. palustris* under different salt concentrations. At higher salt concentrations (2.0 and 2.5%), the strains CGA009-Δ*crtC* and CGA009-Δ*crtF* barely showed any growth, whereas strain CGA009-Δ*crtD* showed a higher growth rate than CGA009. These results suggested that strain CGA009-Δ*crtD* exhibited higher salt tolerance, while strains CGA009-Δ*crtC* and CGA009-Δ*crtF* showed lower tolerance than that of wild-type CGA009. Lycopene desaturase (CrtI) converts phytoene to lycopene through four desaturation steps. CrtC catalyzes the hydration reaction to convert lycopene into rhodopin, which in turn saturates to 3,4-dihydrorhodopin under the catalysis of CrtD. 3,4-dihydrorhodopin can subsequently undergo methylation reaction under the catalysis of *crtF* and become anhydrohodovibrin (Figure 4A). Theoretically, strain CGA009-Δ*crtC*, CGA009-∆*crtD*, and CGA009-Δ*crtF* can accumulate lycopene, rhodopin and 3,4-dihydrorodopine, respectively (Takaichi, 1999). Therefore, it was reasonable to say that, among the carotenoids, rhodopin played a key role in salt tolerance. These results were backed by the findings of RT-qPCR, where a reduction in *crtD* expression was detected in RPAS-11, in response to salt stress. To further illustrate the effect of rhodopin on salt tolerance, *crtC* was overexpressed in CGA009, obtaining strain CGA009-CrtC. However, at higher salt concentrations (2.0 and 2.5%), the growth of strain CGA009-CrtC has no significant difference with the control strain CGA009-pBBRkz (Supplementary Figure S3). It seems that the wild-type CGA009 has different salt-tolerant mechanism with RPAS-11. In the strain RPAS-11, high salt stress would cause ROS production and carotenoid accumulation. And accumulation of carotenoids, especially rhodopin, was speculated to help RPAS-11 to tolerate salt stress. The wild-type CGA009 lacks the potential mutations like RPAS-11 that cause salt tolerance mechanisms. Therefore, overexpression of *crtC* did not lead to better salt tolerance in CGA009 (Supplementary Figure S3). The other possible reason was that single overexpression of *crtC* might not lead to increase in rhodopin content, and overexpression of *crtC* would consume the cell energy for cell growth. In conclusion, carotenoid synthesis, especially rhodopin, plays a role in the salt tolerance ability of *R. palustris*.

**Table 5:** Generation time of *R. palustris* strains with *crtC*, *crtD*, or *crtF* deletion under salt stress.

	Generation time (h)			
Salt concentration	CGA009	CGA009-∆*crtC*	CGA009-∆*crtD*	CGA009-∆*crtF*
2.0%	48.09 ± 0.17	174.8 ± 0.22	38.05 ± 0.26	85.76 ± 0.13
2.5%	148.9 ± 0.21	626.6 ± 0.11	91.46 ± 0.23	463.3 ± 0.24

Symbol ± indicated the standard value.

### 3.4 Salt stress promotes lycopene production in *Rhodopseudomonas palustris*

**Figure 8:**
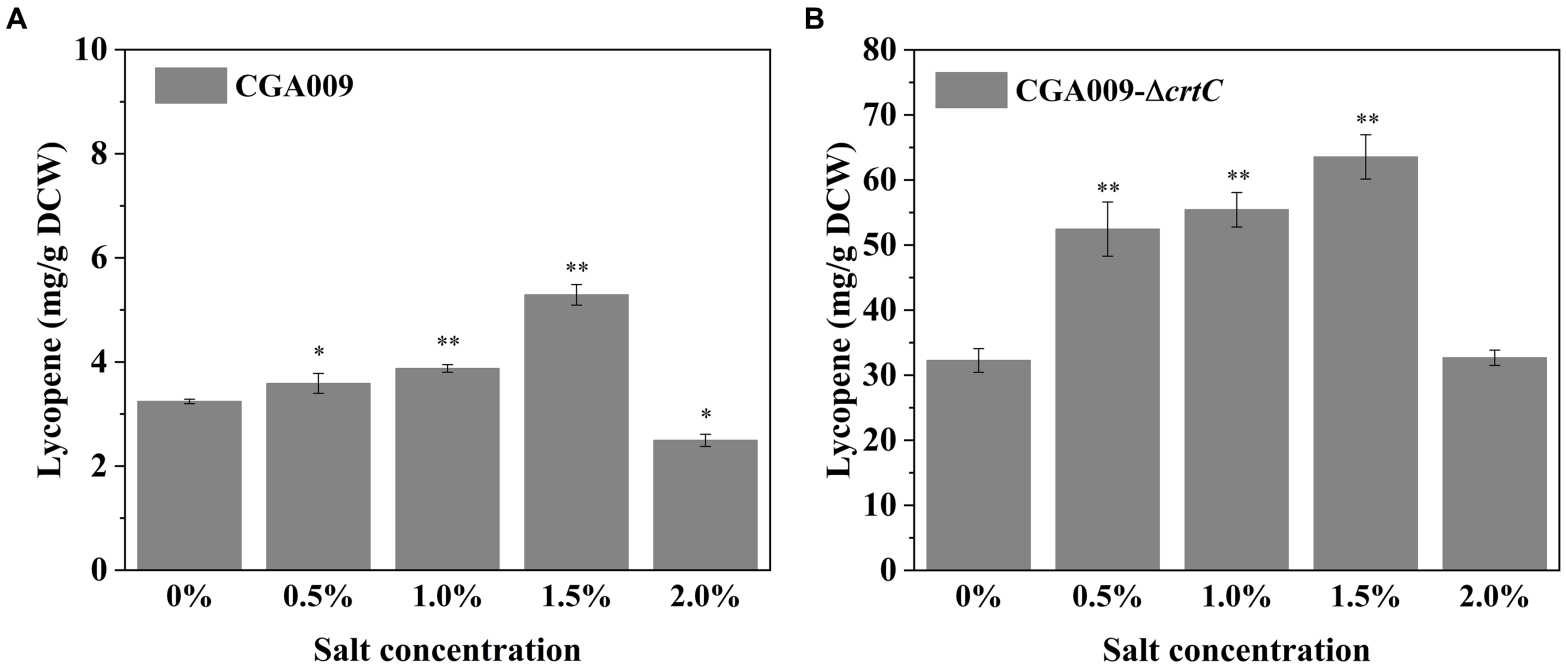
Lycopene content of CGA009 and CGA009-Δ*crtC* under salt stress. **(A)** Lycopene content of CGA009 under salt stress. **(B)** Lycopene content of CGA009-Δ*crtC* under salt stress. Strains were cultivated in a PM medium containing 20 mM acetate as the carbon source and different salt concentrations (0, 0.5, 1.0, 1.5, and 2.0%). Three biological replicates were performed for each treatment. Error bars: mean ± standard deviation (*n* = 3). *p < 0.05 was considered to be statistically significant. **p < 0.01. *p* value was calculated by Excel.

In the present study, the effect of salt stress on lycopene accumulation in *R. palustris* (Li et al., 2022b). Lycopene production in wild-type CGA009 and CGA009-Δ*crtC* knockout *palustris* was investigated. To improve lycopene production, CGA009-Δ*crtC* knockout was constructed to block the carotenoids synthetic pathway, consequently accumulating lycopene strains grown at different salt concentrations (0, 0.5, 1, 1.5, and 2%) was measured (Figure 8). In wild-type CGA009, the lycopene production was increased from 3.24 mg/g DCW to 5.29 mg/g DCW with an increase in salt concentration from 0 to 1.5% (Figure 8A). In *crtC* knockout, CGA009-Δ*crtC*, the lycopene production was increased from 32.26 mg/g DCW to 63.53 mg/g DCW with an increase in salt concentration from 0 to 1.5% (Figure 8B). These results indicated that salt stress stimulated production of lycopene in *R. palustris*. However, at the higher salt concentration (2%), the lycopene production in CGA009 and CGA009-Δ*crtC* was 2.49 mg/g DCW and 32.67 mg/g DCW, respectively. Based on this, it was speculated that higher salt concentration (2%) might have harmed the normal metabolism in *R. palustris*, leading to decreased lycopene production. As a result, salt stress could stimulate lycopene production in *R. palustris* under salt concentrations lower than 2%.

## 4 Discussion

Due to its metabolic versatility and carbon source diversity, *R. palustris* has great potential in wastewater treatment and the production of valuable compounds. However, most kinds of *R. palustris* belong to freshwater bacteria, whose application in high salinity conditions is limited. In this study, salinity-evolved strains were screened, and the relationship between salt tolerance and carotenoid production was investigated.

Adaptive laboratory evolution was used to obtain RPAS-11 strain with an ability to grow at 4.5% salt concentration which is higher than the average seawater salinity of 3.5%. The selected RPAS-11 exhibited relatively higher salt tolerance than that of its ancestral strain CGA009. Further, the selected RPAS-11 showed increased salt tolerance with different carbon sources, including sodium succinate, sodium malate, sodium acetate, and sodium malonate, which mainly exist in industrial and agricultural wastewater (Li et al., 2022a). Our results displayed the potential application of salt-tolerant RPAS-11 in highly-saline wastewater treatment.

In comparison to CGA009, RPAS-11 displayed a significant change in color, indicating a change in carotenoid synthesis under salt stress. Therefore, the possible association between carotenoid production and salt tolerance was further analyzed. With an increase in salt concentration, a reduction in carotenoid production was recorded in wild-type CGA009, whereas an increase was noticed in the case of salt-tolerant RPAS-11. In turn, the positive effect of carotenoid accumulation on salt tolerance was further demonstrated. In strain RPAS-11-IDI, an overexpression of IDI resulted in an increased carotenoid accumulation and high salt tolerance. On the contrary, the knockout strain CGA009-Δ*crtI* showed decreased salt tolerance in the absence of carotenoid synthesis. Likewise, the salt and drought stress in *Nicotiana tabacum* induced the tobacco lycopene β-cyclase gene *Ntβ-LCY1*, further enhancing the expression of carotenoid synthetic genes and carotenoid content (Shi et al., 2015).

Likewise, the relationship between carotenoids and salt tolerance has been studied in some other organisms. Carotenoids are considered as strong antioxidant which can improve the fluidity of the cell membrane and protect the cells from osmotic stress and oxidative damages (D'Souza et al., 1997; Camacho-Córdova et al., 2014). Salt stress was usually considered to stimulate carotenoids production. In the salt-tolerant RPAS-11, increase in carotenoid production was obtained under salt stress (2%). In the case of certain microalgae, moderate salt stress was noticed to induce the accumulation of carotenoids, which further acted as antioxidants and cell protectants, thereby increasing the likelihood of microalgae survival (Giani et al., 2021; Matarredona et al., 2021; Ren et al., 2021). However, for *Haloferax mediterranei*, known for its tolerance to extreme salt levels, the optimal salt concentration for pigmentation accumulation was observed at 12.5% w/v, and decreased carotenoids production was observed with increased salt concentrations (>12.5% w/v), which might due to that for hyper-halophilic strain, low concentration might also be a stress (Thombre et al., 2016). For another kind of hyper-halophilic archaeon *Haloarcula marismortui* RR12, similar relationship between carotenoids production and salt concentration was obtained, and the optimal salt concentration for red carotenoid production was 25% w/v. Furthermore, high salt stress which exceeds the tolerance range would affect the cell growth and carotenoids. In non-salt resistant *Bixa orellana* L., salt stress greatly affected the photosynthetic machinery by reducing the accumulation of chlorophyll pigments and carotenoids (Sankari et al., 2019). In the wild-type CGA009, the carotenoids production was decreased when the salt concentration was increased to 1 and 2%. The osmoprotective effect of another kind of isoprenoid, ubiquinones, have also been researched. In *E. coli*, isoprenoid ubiquinone-8 accumulation improves osmotic-stress tolerance by stabilizing the cell membrane (Sévin and Sauer, 2014). Ubiquinone-10 can modulate the mechanical strength and permeability of lipid membranes, and it is considered as a powerful antioxidant (Agmo Hernández et al., 2015). Of course, the salt tolerance of *R. palustris* may not be solely related to its carotenoid synthesis. It is well known that the regulation of cellular metabolism is a complex process, and many factors may jointly affect its salt tolerance, such as sodium efflux pump, accumulation of compatible solutes, the composition of the cell wall and membrane and so on (Hagemann, 2011; Dakal et al., 2014; Verma et al., 2019). However, it is undeniable that the synthesis of carotenoids is important for the salt tolerance of *R. palustris*.

In *R. palustris*, several pigments, including lycopene, anhydro rhodovibrin, rhodovibrin, rhodopin, and spirilloxanthin were produced, and the key pigment related to salt tolerance was further identified. The RT-qPCR analysis of RPAS-11 revealed that *crtD* was barely expressed under 2% salt stress, which might block the subsequent reactions of carotenoid synthesis and lead to the accumulation of rhodopin. Besides, the knockout strain CGA009-Δ*crtD* not only exhibited relatively better salt tolerance than CGA009, but also displayed salt tolerance at higher salt concentrations (2.0 and 2.5%). These results suggested that the acquired salt tolerance in RPAS-11 might be related to the accumulation of carotenoids, particularly rhodopin. To the best of our knowledge, the promoting effect of rhodopin on salt tolerance was speculated for the first time in the present study. Besides, the effect of high light irradiance on rhodopin content was also reported in *R. palustris* 42OL, where an increase in the percent of rhodopin among the total carotenoids was noticed under aerobic and H_2_-producing conditions (Muzziotti et al., 2017). Moreover, the antioxidant activity of rhodopin in marine seaweeds has already been established (Mohy and Elahwany, 2015; Ashour et al., 2020). These results indicated that antioxidant rhodopin confers tolerance to salt and high light.

Lycopene is a carotenoid with antioxidant, anticancer, and anti-inflammatory properties with numerous applications in the pharmaceutical, cosmetic, and food industries (Li et al., 2020). The production of lycopene using *R. palustris* has already been reported (Niedzwiedzki et al., 2007; Muzziotti et al., 2017). Lycopene generation is one of the physiological responses to stress conditions. Both CGA009 and CGA009-Δ*crtC* showed an increase in lycopene production at low salt concentrations, 0.5, 1.0, and 1.5% (Figure 8). However, the overall carotenoids content in CGA009 registered a decline at 1.0% salt concentration (Figure 4B). This phenomenon indicated a change in the percentage distribution of carotenoids, at 1.0% salt concentration. A previous study has also reported an enhancement in lycopene production in *Blakeslea trispora*, under cold stress conditions (Lingran et al., 2019). Another study reported enhanced synthesis of β-carotene (another carotenoid compound) in *Dunaliella salina*, a halophyilic bacteria that grows well in higher salinity environments, in an environment with salt concentrations lower than optimal for growth, where the highest amounts of β-carotene were obtained at 2.5 mol/L salinity (Hashemi et al., 2020).

In the present study, a salt-tolerant *R. palustris* strain RPAS-11 was obtained through the adaptive laboratory evolution method. The improvement in salt tolerance was evaluated in relation to the carotenoid production in the RPAS-11. The results suggested that salt stress could induce carotenoid production in salt-tolerant strains, and in turn, carotenoid accumulation could help the cells to tolerate salt stress. Further, rhodopin was presumed to be the key pigment related to salt tolerance in *R. palustris* for the first time. The enhancement of salt stress resulted in increased production of another carotenoid compound, lycopene. To sum up, our research provides insight into the salt tolerance mechanism of *R. palustris* and also provides the theoretical basis for the application of *R. palustris* in highly saline wastewater treatment and valuable chemicals production.

## Data availability statement

The original contributions presented in the study are included in the article/Supplementary material, further inquiries can be directed to the corresponding authors.

## Author contributions

MEL: Writing – original draft, Writing – review & editing. TZ: Writing – original draft, Writing – review & editing. RY: Writing – review & editing. ZW: Writing – review & editing. MIL: Writing – review & editing. JY: Writing – review & editing.
