# Supplementary material for: Carotenoids synthesis affects the salt tolerance mechanism of Rhodopseudomonas palustris
Source: Front Microbiol. 2023 Nov 22;14:1292937. doi: 10.3389/fmicb.2023.1292937 (PMC10702980; doi:10.3389/fmicb.2023.1292937)
Supplement: Supplementary file 1 [file Data_Sheet_1.docx]

Supplementary Material

# Supplementary Figures and Tables

## Supplementary Figures

**
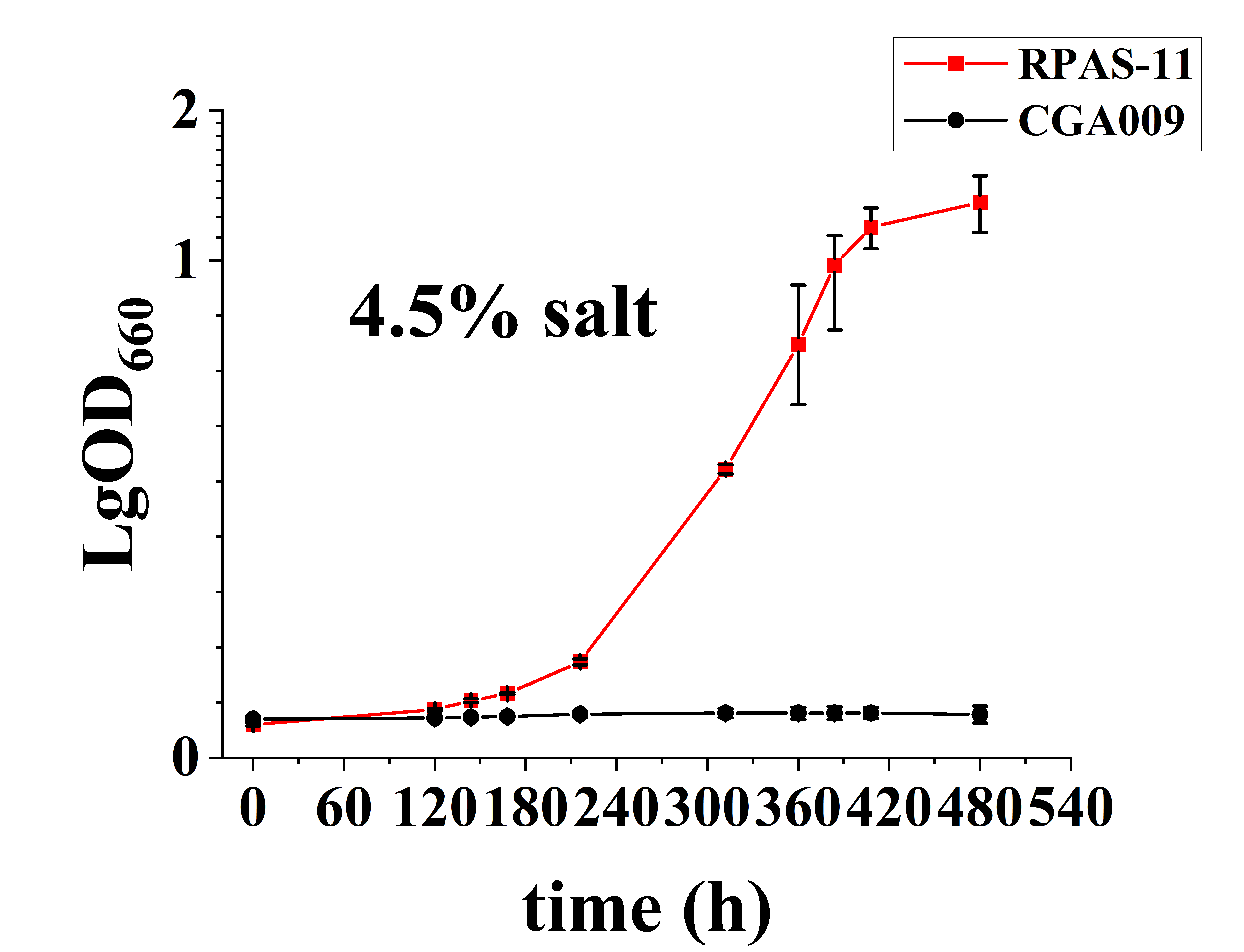
**

**Supplementary Figure S1.** Growth curves of RPAS-11 and CGA009 at 4.5% salt concentration. RPAS-11 and CGA009 were cultivated in PM medium with 20 mM acetate as the carbon source at 4.5% salt concentration. Three biological replicates were performed.

**
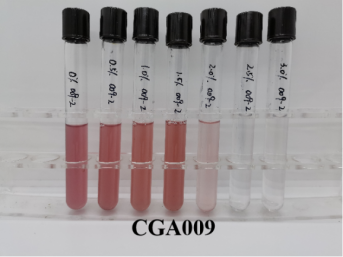

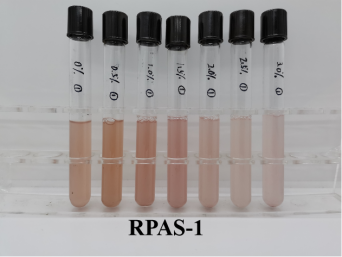

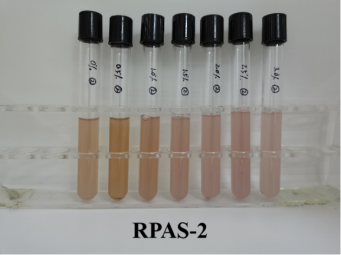

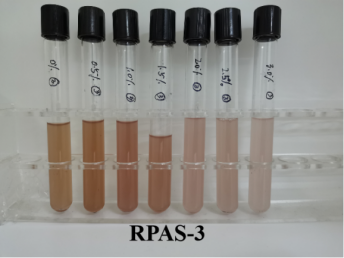

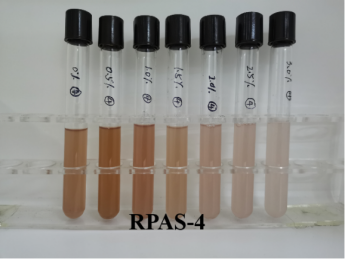

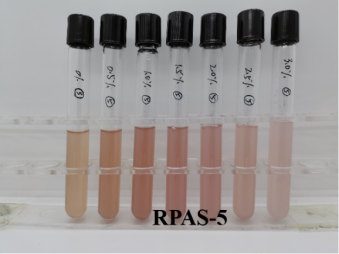

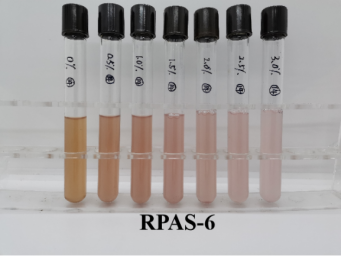

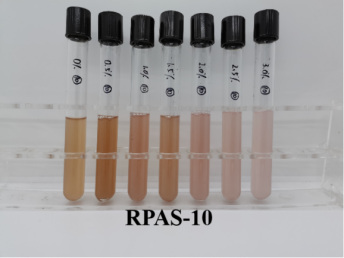

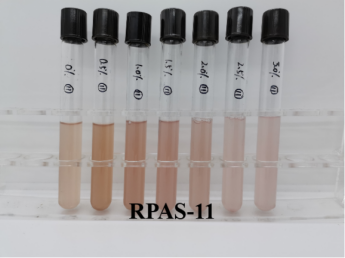
**

**Supplementary Figure S2.** Color of *R. palustris* strains at different salt concentrations. Strains were cultivated in PM medium with 20 mM acetate as the carbon source at different salt concentrations (0%, 0.5%, 1.0%, 1.5%, 2.0%, 2.5% and 3.0%).

**
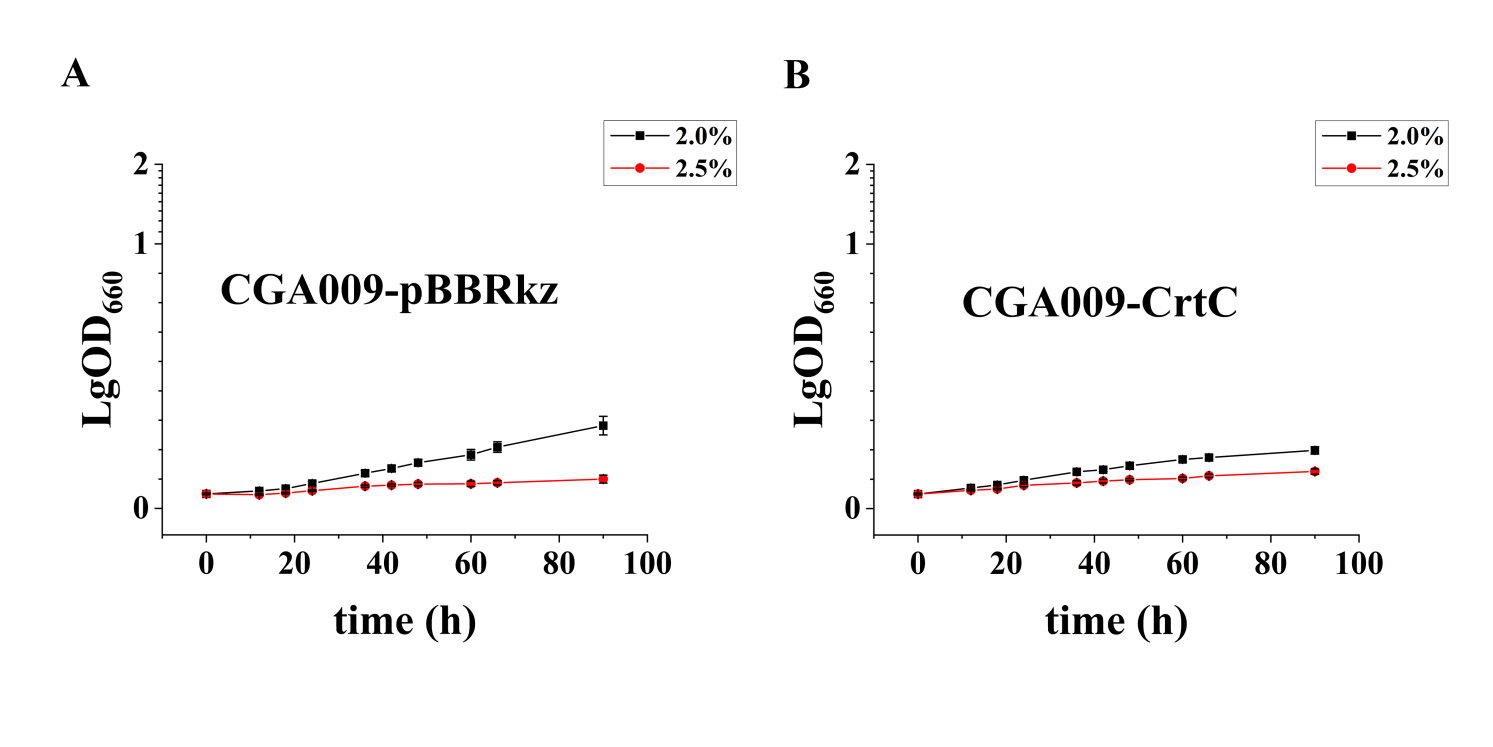
Supplementary Figure S3.** Growth curves of CGA009 strain overexpressing *crtC* and wild-type CGA009 under salt stress. Strains were cultivated in PM medium with 20 mM acetate as the carbon source at different salt concentrations (2.0% and 2.5%).

**1.2 Supplementary Tables**

**Supplementary Table S1.**  Primers used for real time qPCR analysis

| Primer | Sequence (5’-3’) |
| --- | --- |
| crtB-F | GCAATCCGATATGTTGGCCC |
| crtB-R | CTTGGAGGCAGCGTGAAAGG |
| crtI-F | TGGTGATCGGCTCTGGTTTC |
| crtI-R | TCGAATGAGAAGCCGTCCTG |
| crtC-F | GGCGTAGAACGGAGTGTCTT |
| crtC-R | GCCGAAGGTAACAAGCAGGA |
| crtD-F | CAGACATACACGGTCGGCTC |
| crtD-R | AAACTGAGGGCTTCCCACTG |
| crtF-F | TCTCATTGAGCCTGCGTGAC |
| crtF-R | GACGCATCAACGGAAACACC |

**Supplementary Table S2.** The primers utilized for suicide plasmid construction and mutant strain verification

| Primer | Sequence (5’-3’) |
| --- | --- |
| ΔcrtI-UP-F | TCGAATTCCTGCAGCCCGGGGGATCCACCTGGTCTGGAGTCGCGAT |
| ΔcrtI-UP-R | GCCAGGATCGAGCATGGGTACCACTGTAACAAAAAAT |
| ΔcrtI-DOWN-F | CCCATGCTCGATCCTGGCGTCTGCAATCCGATATGTTGGCC |
| ΔcrtI-DOWN-R | CGCTCTAGAACTAGTGGATCCGGCAGTTCGCGCACCGGATG |
| pJQ200SKzt-F | GCCGGATCCACTAGTTCTAGAGCGG |
| pJQ200SKzt-R | CCCCGGGCTGCAGGAATTCGATA |
| pJQ-YZ-F | ATTGCCGCCCTGATCGAACG |
| pJQ-YZ-R | CGGGCGCAATACGAATACAG |
| crtI-YZ-R | AGCAATGTGCGGAACTCGGCACGCA |
| crtI-YZ-F | GCCGAAGATTTCCGGATGGCTGCTG |
| Genome-I-F | TGACCAAGGCCGGGACCTGA |
| Genome-I-R | TTCGAGGCGGGTGAACAGGT |
| crtC-F | GCGGCCGCTCTAGAACTAGTCTACCTCCGCCGCGGCATCC |
| crtC-R | TTCCTGCAGCCCGGGGGATCCATGATTGCCTGGCCTAACG |
| pBBRzt-F | GGATCCCCCGGGCTGCAGGAATTC |
| pBBRzt-R | CCCCCGGGCTGCAGGAATTC |
| crtC-YZ-F | CGCTTACAATTTCCATTCGCCATTCAGGCT |
| crtC-YZ-R | GTGAGTTAGCTCACTCATTAGGCACCCCA |
